# Isolated diastolic hypertension and cardiovascular outcomes across different diagnostic guidelines: a systematic review and meta-analysis

**DOI:** 10.1186/s43044-024-00556-5

**Published:** 2024-09-14

**Authors:** Abhimanyu Agarwal, Mohamed A. Mostafa, Muhammad Imtiaz Ahmad, Elsayed Z. Soliman

**Affiliations:** 1 Section on Cardiovascular Medicine, Department of Medicine, Epidemiological Cardiology Research Center (EPICARE), Wake Forest University School of Medicine, Medical Center Blvd, Winston-Salem, NC 27157 USA; 2 Section on Hospital Medicine, Department of Internal Medicine, Medical College of Wisconsin, Wauwatosa, WI USA

**Keywords:** IDH, CVD, ACC/AHA, JNC7, NICE/ESC

## Abstract

**Background:**

This systematic review aims to determine the impact of isolated diastolic hypertension (IDH) on cardiovascular outcomes.

**Methods:**

We searched only English language articles on PubMed and SCOPUS until July 31, 2023 to investigate the association between IDH and cardiovascular outcomes.

**Results:**

This meta-analysis of 19 studies evaluated the impact of different hypertension diagnostic guidelines (ACC/AHA: American Heart Association/American College of Cardiology; JNC7: Joint National Committee on Prevention, Detection, Evaluation, and Treatment of High Blood Pressure; NICE/ESC: National Institute for Health and Care Excellence/European Society of Cardiology) on hypertension-related outcomes. Studies had varying sample sizes (173 to 2,969,679 participants) and study designs. In cohort studies using JNC7 guidelines, IDH was linked to increased cardiovascular disease (CVD) risk (HR: 1.45, 95% CI 1.17, 1.74), CVD mortality (HR: 1.54, 95% CI 1.23, 1.84), and coronary heart disease (CHD) risk (HR: 1.65). In studies using ACC/AHA guidelines, associations with CVD risk and CVD mortality were weaker [HR: 1.16 (95% CI 1.06, 1.25) and 1.10 (95% CI 0.95, 1.25), respectively]. Subgroup analysis revealed differences in outcomes on the basis of age and sex. Cross-sectional studies did not show significant associations with JNC7 and ACC guidelines; NICE guidelines were not used in cross-sectional studies.

**Conclusion:**

IDH is associated with an increased risk of CVD. Higher diastolic blood pressure cutoffs were associated with higher cardiovascular risk. This association varied by study design and effect modification by sex and race influenced the association.

## Background

Hypertension presents a significant risk factor closely associated with the morbidity and mortality of cardiovascular disease (CVD), exerting a profound impact on public health [1]. Within the spectrum of hypertension, a noteworthy subtype that warrants attention is isolated diastolic hypertension (IDH). This particular subtype is defined as systolic blood pressure (SBP) below 130 mm Hg and a diastolic blood pressure (DBP) of at least 80 mm Hg or higher, according to the criteria established by the American College of Cardiology (ACC) and the American Heart Association (AHA) [2] in 2017. Alternatively, the criteria outlined by the European Society of Cardiology (ESC) [3] in 2018 and the Joint National Committee on Prevention, Detection, Evaluation, and Treatment of High Blood Pressure (JNC7) [4] guidelines define IDH as an SBP below 140 mm Hg and a DBP of 90 mm Hg or higher.

Examining the trajectory of hypertension development reveals a compelling pattern: while diastolic blood pressure typically increases progressively from childhood to around the age of 50, thereafter, systolic blood pressure becomes more prominent, primarily due to arteriosclerosis affecting aging arteries. Consequently, complications related to hypertension and cardiovascular issues predominantly arise from elevated systolic blood pressure in older individuals [5, 6]. Interestingly, this suggests that high diastolic pressure is more common among younger and middle-aged populations [7–10]. Delving into the demographics of isolated diastolic hypertension (IDH) reveals intriguing nuances. It appears to occur slightly more frequently in men than in women [10, 11]. Furthermore, its correlation with central obesity, intertwined with other components of the metabolic syndrome, is particularly prevalent among young individuals. This intriguing association suggests a potential link between lifestyle factors and the emergence of IDH [10, 12, 13].

Given these observations, this study explores the complexities of isolated diastolic hypertension (IDH), aiming to uncover its underlying dynamics and consequences. By understanding the unique features of this subtype, we can potentially identify new strategies for managing and reducing the widespread influence of hypertension on public health worldwide.

## Methods

We conducted a systematic review and meta-analysis to examine the relationship between isolated diastolic hypertension (IDH) and cardiovascular complications. Our methodology followed the guidelines specified in the Preferred Reporting Items for Systematic Reviews and Meta-Analyses (PRISMA) [14].

### Data sources

We performed a systematic search of the PubMed and SCOPUS databases from their inception until July 31, 2023. Our search terms included 'hypertension,' 'high blood pressure,' 'isolated diastolic hypertension,' 'IDH,' 'cardiovascular diseases,' 'cardiovascular events,' 'cardiovascular mortality,' 'myocardial ischemia,' 'coronary artery disease,' 'coronary heart disease,' 'ischemic heart disease,' 'myocardial infarction,' 'chronic heart failure,' 'stroke,' 'ischemic stroke,' 'hemorrhagic stroke,' 'cerebrovascular disorders,' 'cerebrovascular events,' 'cerebrovascular mortality,' and 'cerebrovascular death.' Additionally, we manually reviewed the reference lists of pertinent articles to find additional studies.

#### Eligibility criteria

We included only studies published in English that met the following criteria:

- Observational studies or randomized controlled trials
- Studies involving patients with isolated diastolic hypertension (IDH)
- Studies reporting the outcome of interest

#### Exclusion criteria

We excluded studies focused on the pathophysiology of IDH and cardiovascular complications, studies involving critically ill patients, and studies that did not report data on cardiovascular complications or only provided biomarkers or genetic markers. Additionally, studies that did not present original data, such as editorials, case reports, case series, systematic reviews, or meta-analyses, were excluded. Based on these criteria, we assessed the eligibility of the included studies.

### Study identification

We screened articles based on their titles and abstracts using predefined inclusion and exclusion criteria along with a standardized data form. Full-text articles were not reviewed if they did not meet the inclusion criteria based on the abstract. Decisions regarding the inclusion of full-text articles were reached by consensus. All findings were imported into Zotero, an open-source research tool used for organizing and analyzing data, where duplicate entries were removed.

### Data extraction and outcomes

We utilized a structured data collection form to compile information from each study, including study design, patient characteristics, baseline variables, duration of follow-up in years, identification of isolated diastolic hypertension (IDH) according to various guidelines such as ACC/AHA 2017 (American Heart Association/American College of Cardiology), JNC7 (Joint National Committee on Prevention, Detection, Evaluation, and Treatment of High Blood Pressure), NICE/ESC (National Institute for Health and Care Excellence/European Society of Cardiology), maximum adjusted covariates, and adjusted hazard ratio (HR), relative risk (RR), or odds ratio (OR) with corresponding 95% confidence intervals (CI). In cases where duplicate studies were identified and both reported the same outcome measure, only the more comprehensive study was included in the analysis.

### Quality assessment

Discrepancies were resolved through consensus. Epidemiological and clinical data from the included studies were extracted using standardized forms. The quality of the articles was assessed using the Newcastle–Ottawa Scale (NOS). Articles scoring ≥ 6 stars on the NOS were considered high quality, while those scoring < 6 stars were considered low quality.

### Data analysis

We gathered baseline characteristics, sample sizes, and adjusted hazard ratios (HR), relative risks (RR), or odds ratios (OR) for both primary and secondary outcomes. All statistical calculations were performed using JASP 0.17.2.1. Significance was determined at a p value of 0.05.

A random-effects model was utilized to accommodate variability within and across studies. The Higgins *I*-squared statistic (*I*^2^) was employed to assess the degree of heterogeneity, with interpretations based on thresholds outlined in the Cochrane Handbook for Systematic Reviews of Interventions:

- 0–40%: may not be significant.
- 30–60%: could indicate moderate heterogeneity.
- 50–90%: may suggest substantial heterogeneity.
- 75–100%: indicates considerable heterogeneity.

Publication bias was assessed using funnel plots and quantified through Egger's regression test. If publication bias was detected, subsequent trim-fill analysis was conducted to adjust for this bias. A forest plot was used to illustrate the magnitude of impact in each study and the combined estimate. Additionally, a subgroup analysis was performed to explore potential effect modifications by age, sex, and study design (cohort vs. cross-sectional).

## Results

**Fig. 1:**
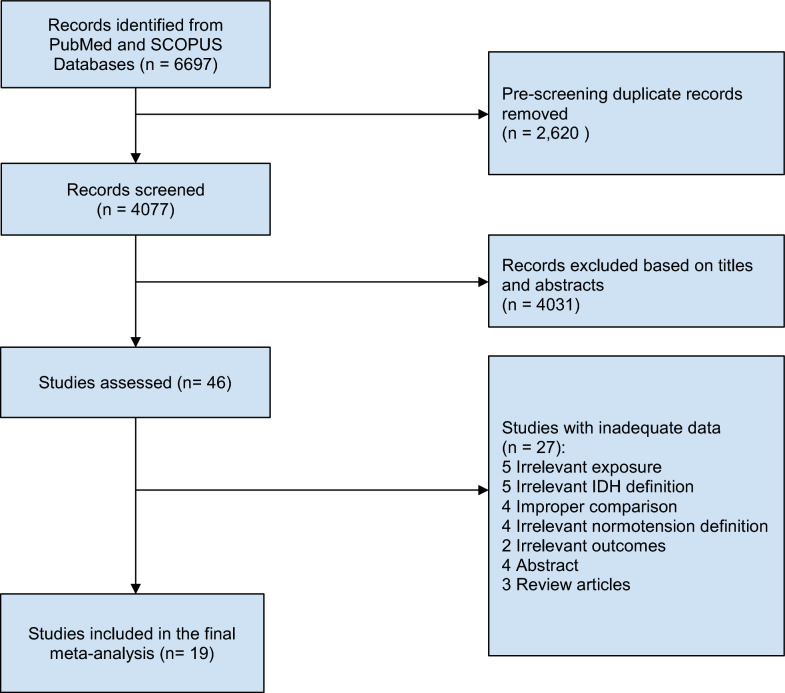
Study selection using PRISMA technique

This meta-analysis comprised 19 research articles [15–33]. Of these, eight studies [16–18, 21, 24, 29, 32, 33] provided data for two or more guidelines within the same article resulting in 31 datasets for the meta-analysis. Figure 1 illustrates the methodology used to select the studies, while Table 1 provides a summary of the principal characteristics of the included studies.

**Table 1:** Basic characteristics of study population

S. no	Study authors	Total patients	Follow up (years)	Mean age (years)	Men %	Type of study	Location	Guideline used to diagnose IDH
1	Yue et al. [15]	8853	3.20	57.38	38.45	Prospective cohort	China	JNC7
2	Yano et al. [16]	4850	31.10	33.89	100.00	Prospective cohort	USA	JNC7
3	Yano et al. [16]	6263	31.10	31.01	0.00	Prospective cohort	USA	JNC7
4	Fu-Rong et al. [17]	91,303	8.10	51.73	28.52	Prospective cohort	UK	NICE/ESC
5	Fu-Rong et al. [17]	91,303	8.10	51.73	28.52	Prospective cohort	UK	ACC/AHC
6	McGrath et al. [18]	151,831	9.80	54.57	39.69	Prospective cohort	UK	NICE/ESC
7	McGrath et al. [18]	89,126	10.00	53.54	33.67	Prospective cohort	UK	ACC/AHC
8	Shouling et al. [19]	61,961	10.41	48.72	77.25	Prospective cohort	China	JNC7
9	Li et al. [20]	899	9.00	42.61	38.26	Prospective cohort	China	JNC7
10	Lotfaliany et al. [21]	5959	10.06	42.58	44.91	Prospective cohort	Iran	JNC7
11	Lotfaliany et al. [21]	425	10.06	69.82	66.00	Prospective cohort	Iran	JNC7
12	Arima et al. [22]	152,491	7.00	48.00	59.00	Prospective cohort	Asia, Australia, New Zealand	JNC7
13	Lee et al. [23]	2,969,679	13.20	29.31	45.99	Prospective cohort	South Korea	JNC7
14	Zhang et al. [24]	28,375	11.30	48.17	50.78	Prospective cohort	China	JNC7
15	Zhang et al. [24]	19,688	11.30	47.16	47.47	Prospective cohort	China	ACC/AHC
16	Guo et al. [25]	153,152	10.00	47.71	34.24	Prospective cohort	China	JNC7
17	Fang et al. [26]	18,787	9.50	49.12	48.79	Prospective cohort	China	JNC7
18	Kelly et al. [27]	128,752	8.30	54.04	50.13	Prospective cohort	China	JNC7
19	Barengo et al. [28]	13,537	16.00	40.50	NA	Cross-sectional	Finland	JNC7
20	Carlsson et al. [29] (M)	183	26.00	46–65	100.00	Prospective cohort	Sweden	JNC7
21	Carlsson et al. [29] (F)	173	26.00	46–65	0.00	Prospective cohort	Sweden	JNC7
22	Sun et al. [30]	27,579	4.30	48.25	50.73	Prospective cohort	China	JNC7
23	Hisamatsu et al. [31]	1474	29.00	38.15	34.00	Prospective cohort	Japan	JNC7
24	McEvoy et al. [32] (ARIC Study)	10,540	25.20	56.36	43.24	Cross-sectional	USA	JNC7
25	McEvoy et al. [32] (ARIC Study)	8703	25.20	56.00	42.81	Cross-sectional	USA	ACC/AHC
26	McEvoy et al. [32] (NHANES)	34,904	9.80	42.00	NA	Cross-sectional	USA	JNC7
27	McEvoy et al. [32] (NHANES)	29,525	9.80	40.00	NA	Cross-sectional	USA	ACC/AHC
28	McEvoy et al. [32] (CLUE II)	17,654	28.70	45.00	NA	Cross-sectional	USA	JNC7
29	McEvoy et al. [32] (CLUE II)	13,238	28.70	42.00	NA	Cross-sectional	USA	ACC/AHC
30	Jacobsen et al. [33]	5099	13.00	60.46	48.51	Prospective cohort	USA	NICE/ESC
31	Jacobsen et al. [33]	4057	13.00	59.47	48.76	Prospective cohort	USA	ACC/AHC

**Fig. 2:**
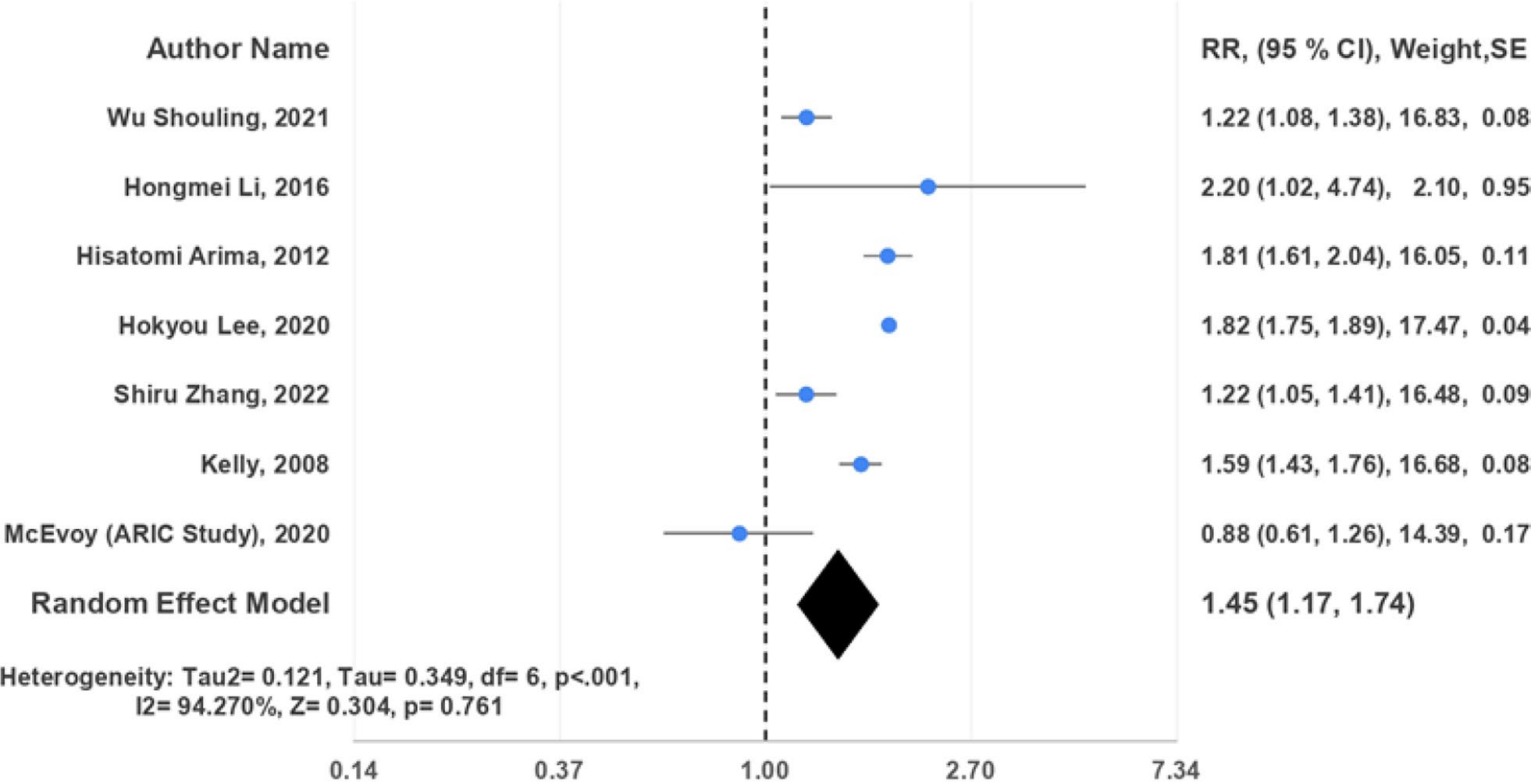
Random effects forest plot for IDH and risk of CVD among studies using JNC7 guidelines

**Fig. 3:**
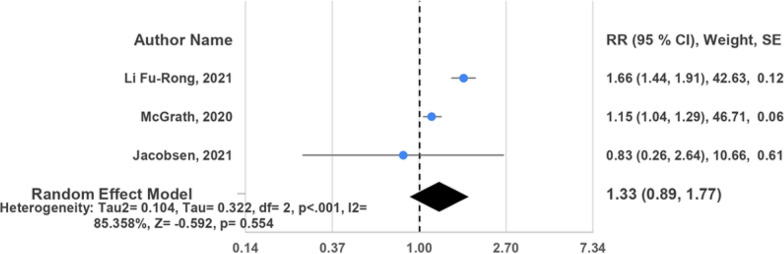
Random effects forest plot for IDH and risk of CVD among studies using NICE guidelines

**Fig. 4:**
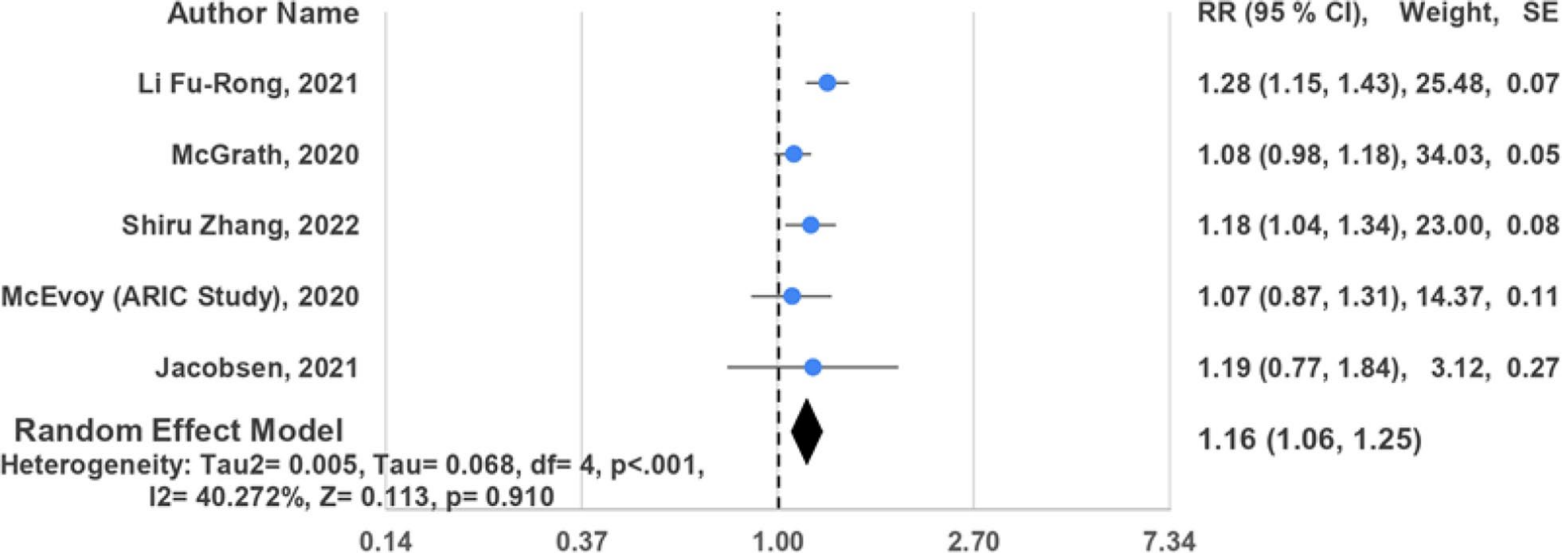
Random effects forest plot for IDH and risk of CVD among studies using ACC guidelines

Patients with IDH exhibited a wide range of cardiovascular outcomes. These included an increased risk of cardiovascular diseases such as myocardial infarction, cerebral infarction, and cerebral hemorrhage. Additionally, there was a heightened risk of cardiovascular disease mortality, coronary heart disease (CHD) and CHD mortality, stroke and its subtypes (hemorrhagic and ischemic), and all-cause mortality. Of these investigations, 17 studies adopted a prospective cohort design [15–27, 29–31, 33], while 2 were cross-sectional analyses [28, 32]. The included studies enrolled between 173 and 2,969,679 participants. Among these studies, the JNC7 guideline [15, 16, 19–32] was the most frequently utilized for diagnosing IDH, followed by ACC/AHA [17, 18, 24, 32, 33] and NICE/ESC [17, 18, 33] guidelines (Figs. 2, 3, 4).

The total number of patients diagnosed with IDH using the JNC7, ACC/AHA, and NICE/ESC guidelines were 3,646,490, 255,640, and 248,233, respectively. Cohort studies comprised 4,022,262 enrolled patients, whereas 128,101 participants were included in cross-sectional studies.

### Quality of studies and publication *bias*

All included studies demonstrated high methodological rigor, with quality scores ranging from 7 to 9 (see supplementary material). To assess potential publication bias in the pooled estimates, we first examined funnel plot asymmetry, visually represented in the supplementary material. Significant publication bias was detected in the pooled HRs for CVD mortality in studies following the ACC/AHA guidelines and for all-cause mortality in studies adhering to the JNC7 guidelines.

We further evaluated publication bias using Egger’s test, with detailed results presented in Table 2. Interestingly, no publication bias was found in the pooled HRs for CVD risk, CHD risk and mortality, or stroke risk in IDH patients, regardless of the guidelines followed. Subgroup analyses also did not reveal any notable publication bias, except for CVD mortality in women and the risk of CVD and all-cause mortality in cohort studies.

**Table 2:** Publication bias assessment using funnel plot and Egger’s test

Groups	Guidelines	Publication bias assessment by funnel plot (yes/no)	Egger’s test (p value)
CVD	JNC7	No	0.761
	ACC	No	0.910
	NICE	No	0.554
CVD mortality	JNC7	No	0.240
	ACC	Yes	0.061
All-cause mortality	JNC7	Yes	0.034
	ACC	No	0.876
Stroke	JNC7	No	0.988
	ACC	No	NA
Ischemic stroke	JNC7	No	0.662
CHD	JNC7	No	0.842
CHD mortality	JNC7	No	NA
*Subgroup analysis*			
Men			
CVD mortality	JNC7	No	0.946
Stroke	JNC7	No	NA
Women			
CVD	JNC7	No	NA
CVD mortality	JNC7	Yes	0.395
Stroke	JNC7	No	NA
Cohort			
CVD	JNC7	No	0.582
	ACC	Yes	0.730
	NICE	Yes	0.554
CVD mortality	JNC7	No	0.518
All-cause mortality	JNC7	Yes	0.093
Stroke	JNC7	No	0.988
Ischemic stroke	JNC7	No	0.662
CHD	JNC7	No	0.842

To address the identified publication bias, we performed a trim-fill analysis, and the adjusted HRs for each relevant analysis are provided accordingly.

### Analysis of individual results

In the 19 studies included, IDH ascertainment was done utilizing varying guidelines. Therefore, we performed a meta-analysis exclusively on those studies that adhered to uniform guidelines.

### Risk of cardiovascular disease in patients with IDH

Despite an elevated risk of cardiovascular disease (CVD) in patients with isolated diastolic hypertension (IDH), the diagnostic guidelines influenced this observation. Seven studies [19, 20, 22–24, 27, 32] adhering to JNC7 guidelines indicated a 45% increased risk of CVD (pooled HR = 1.45, 95% CI 1.17, 1.74, *I*^2^ = 94.270%). Similarly, three studies [17, 18, 33] following NICE/ESC guidelines demonstrated a 33% increased risk (pooled HR = 1.33, 95% CI 0.89, 1.77, *I*^2^ = 85.358%), comparable to the JNC7 findings. To a lesser extent, analyses of five studies [17, 18, 24, 32, 33] adhering to ACC/AHA guidelines showed a 16% increased risk (pooled HR = 1.16, 95% CI 1.06, 1.25, *I*^2^ = 40.272%).

### Cardiovascular mortality in patients with IDH

A pooled analysis of 11 studies [15, 16, 21, 23–25, 27–29, 31, 32] utilizing JNC7 diagnostic guidelines revealed that patients with isolated diastolic hypertension (IDH) had a higher risk of cardiovascular disease (CVD) mortality (pooled HR = 1.54, 95% CI 1.23, 1.84, *I*^2^ = 82.645%) compared to three studies [17, 24, 32] adhering to ACC/AHA guidelines (pooled HR = 1.10, 95% CI 0.95, 1.25, *I*^2^ = 38.606%). To address the observed publication bias in studies using ACC/AHA guidelines, subsequent trim-fill analysis yielded a pooled HR of 1.01 (95% CI 0.85, 1.17).

### All-cause mortality

An extensive examination of five studies [21, 23, 24, 28, 32] utilizing JNC7 guidelines revealed a pooled hazard ratio (HR) of 1.14 (95% CI 0.98, 1.31, *I*^2^ = 76.779%). Notably, there was a significant indication of publication bias according to Egger's test (*p* = 0.034). Subsequent trim-fill analysis, which accounted for this bias, resulted in a slightly reduced pooled HR of 1.09 (95% CI 0.94, 1.25). Conversely, the combined analysis of three studies [24, 32, 33] adhering to ACC/AHA guidelines showed no increased risk of all-cause mortality in patients with isolated diastolic hypertension (IDH) (pooled HR = 0.97 95% CI 0.91, 1.03, *I*^2^ = 0.00%).

### Risk of stroke in patients with IDH

A collective examination of five studies [23, 24, 26, 27, 30] conducted in accordance with JNC7 diagnostic guidelines demonstrated a 71% higher likelihood of stroke risk (pooled HR = 1.71, 95% CI 1.39, 2.04, *I*^2^ = 88.087%). In contrast, the pooled analysis of two studies [18, 24] adhering to ACC/AHA guidelines revealed only a 17% increased risk of stroke (pooled HR = 1.17, 95% CI 1.00, 1.34, *I*^2^ = 0.00%), comparatively lower than that observed with JNC7 guidelines.

### Risk of ischemic versus hemorrhagic stroke

Combining data from three studies [22, 26, 30] conducted under JNC7 guidelines revealed a 93% higher risk of ischemic stroke (pooled HR = 1.93, 95% CI 1.57, 2.29, *I*^2^ = 0.00%). However, there was no statistically significant association found between isolated diastolic hypertension (IDH) and hemorrhagic stroke using JNC7 guidelines.

### Risk of CHD and CHD mortality in patients with IDH

A pooled analysis of three studies [22, 27, 31] conducted under JNC7 guidelines revealed a 65% increased risk of coronary heart disease (CHD) (pooled HR: 1.65, 95% CI 1.40, 1.90, *I*^2^ = 0.00%). Similar findings were observed for CHD mortality in one study [16] using JNC7 guidelines, which compared the hazard ratios for men and women (pooled HR = 1.63, 95% CI 1.15, 2.10, *I*^2^ = 0.00%).

### Subgroup analysis

*Age*: Lotfaliany et al. [21] conducted a single study that compared the correlation between isolated diastolic hypertension (IDH) and all-cause mortality across two separate age brackets (45 to < 65 and > 65). The findings revealed a more robust association between IDH and all-cause mortality in older patients (HR = 3.23, 95% CI 1.46, 7.16) in contrast to middle-aged individuals (HR = 2.01, 95% CI 1.11, 3.65).

#### Sex

*Men* In a pooled analysis of two studies [22, 30] conducted under JNC7 guidelines, there was no statistically significant association observed with cardiovascular disease (CVD) risk. However, separate analyses of studies adhering to JNC7 guidelines revealed higher CVD mortality [16, 27, 29] (HR = 1.42, 95% CI 1.15, 1.70, *p* < 0.001, *I*^2^ = 23.477%) and stroke risk [26, 27] (HR = 1.88, 95% CI 1.66, 2.10, *p* < 0.001, *I*^2^ = 0.00%) in men.

*Women* In women, a positive association was noted between isolated diastolic hypertension (IDH) and cardiovascular disease (CVD) risk across two studies [19, 27] based on JNC7 guidelines (pooled HR = 1.83, 95% CI 1.54, 2.12, *p* < 0.001, *I*^2^ = 0.00%). However, regarding CVD mortality, the analysis encompassing these guidelines involved three studies [16, 27, 29] and yielded inconclusive results (pooled HR = 1.40, 95% CI 0.80, 2.00, *p* < 0.001, *I*^2^ = 50.312%). Although the funnel plot indicated apparent publication bias, this finding was not supported by Egger’s test (*p* = 0.395). To address this, trim-fill analysis was conducted, resulting in a pooled HR of 1.71 (95% CI 1.09, 2.33). Lastly, the combined analysis of two studies utilizing JNC7 guidelines demonstrated an increased risk of all stroke associated with women (pooled HR = 1.87, 95% CI 1.65, 2.09, *I*^2^ = 0.00%).

### Study design

#### Cohort

*CVD and mortality* Combining six studies [19, 20, 22–24, 27] under the JNC7 guidelines revealed a positive association with the risk of cardiovascular disease (CVD) (pooled HR = 1.54, 95% CI 1.29, 1.80, *I*^2^ = 92.602%). Similarly, applying the ACC guidelines in four studies [17, 18, 24, 33] yielded comparable results (HR = 1.17, 95% CI 1.06, 1.28, *I*^2^ = 49.413%). Notably, although the funnel plot displayed slight publication bias, this was not supported by an Egger's test p value of 0.730. To address this, a trim-fill analysis was conducted, resulting in a pooled HR of 1.12 (95% CI 0.99, 1.24). In contrast, within the NICE guidelines, a synthesis of three studies [17, 18, 33] resulted in a pooled HR of 1.33 (95% CI 0.89, 1.77, *I*^2^ = 85.358%). Similar to the ACC guidelines, the funnel plot displayed mild publication bias, while the p value from Egger's test was 0.554. Trim-fill analysis yielded a pooled HR of 1.39 (95% CI 0.99, 1.79). Regarding CVD mortality, a pooled analysis of nine studies [15, 16, 21, 23–25, 27, 29, 31] using JNC7 guidelines showed a pooled HR of 1.60 (95% CI 1.30, 1.91, *I*^2^ = 73.358%).

*All-cause mortality* Pooling data from three studies [21, 23, 24] under JNC7 guidelines revealed a pooled hazard ratio of 1.20 (95% CI 1.16, 1.25, *I*^2^ = 0.028%). While visual examination of the funnel plot suggested a mild publication bias, this was not supported by Egger’s test (*p* = 0.093). To address this, a trim-fill analysis was conducted, resulting in a pooled hazard ratio of 1.20 (95% CI 1.13, 1.26).

*Risk of stroke* Pooled analysis of five studies [23, 24, 26, 27, 30] using JNC7 guidelines showed a pooled HR of 1.71 (95% CI 1.39, 2.04, *I*^2^ = 88.087%).

*Risk of ischemic and hemorrhagic stroke* A combined analysis of three studies [22, 26, 30] conducted under JNC7 guidelines revealed a pooled hazard ratio of 1.93 (95% CI 1.57, 2.29, *I*^2^ = 0.00%) for ischemic stroke. However, there was no statistically significant association observed for hemorrhagic stroke risk using JNC7 guidelines.

*Stroke mortality* Pooled analysis of two studies [16, 31] using JNC7 guidelines showed no statistically significant association.

*Risk of CHD*: Pooled analysis of three studies [22, 27, 31] using JNC7 guidelines showed a pooled HR of 1.65 (95% CI 1.40, 1.90, *I*^2^ = 0.00%).

*Cross-sectional* Cross-sectional investigations following JNC7 and ACC guidelines revealed no statistically significant results across all measured outcomes. None of the cross-sectional studies used NICE guidelines.

## Discussion

The profound impact of elevated blood pressure, or persistent hypertension, is undeniable. Serving as the primary risk factor for myocardial infarction, stroke, and vascular diseases, hypertension undeniably holds a pivotal position in the global disease landscape [34]. International, national, and regional efforts are currently underway to improve blood pressure control and mitigate the consequent disease burden [35].

Notably, recent updates to guidelines have significantly influenced specific subgroups within the realm of isolated diastolic hypertension (IDH) and isolated systolic hypertension (ISH). This evolution has led to the identification of novel IDH/ISH patient categories across various geographical regions. Importantly, the manifestation of IDH and ISH in younger patients is characterized by distinct population distributions, pathogenic mechanisms, and risk profiles. This divergence in presentation offers an intriguing avenue for investigating the multifaceted causes of hypertension, a condition that has remained elusive in over 90% of hypertensive patients [36].

One of the pivotal findings from this analysis is the notable disparity in outcomes depending on the guidelines utilized for hypertension diagnosis. This underscores the significance of employing standardized criteria when examining the health implications of hypertension. Notably, studies adhering to JNC7 guidelines consistently revealed a heightened risk for cardiovascular disease (CVD), CVD mortality, ischemic stroke, coronary heart disease (CHD) risk, and CHD mortality compared to those utilizing ACC/AHA guidelines. Conversely, studies following NICE/ESC guidelines yielded mixed results, demonstrating an increased risk for CVD but nonsignificant risks for other outcomes, including stroke and CHD.

A subgroup analysis based on sex and study design uncovered intriguing nuances. Among men, studies employing JNC7 guidelines exhibited a higher risk for CVD mortality and all-cause mortality. Conversely, women displayed an elevated risk for CVD and all-stroke mortality, with a mixed pattern observed for CVD mortality. Cohort studies consistently indicated an elevated risk for CVD and CVD mortality across various guidelines. However, the findings from cross-sectional studies were less conclusive, and the utilization of ACC or NICE guidelines was limited. Additionally, some cross-sectional studies indicated a mild publication bias, which could potentially influence the outcomes.

Similar results were reported in a meta-analysis conducted by Huang et al. [37], which demonstrated a significant association between isolated diastolic hypertension (IDH) and an increased risk of composite cardiovascular disease (CVD) and CVD mortality, as well as stroke risk. However, no significant correlation was found between IDH and all-cause mortality. Subgroup analyses revealed consistent associations across most categories, except for older participants and specific regions. Their findings also suggest that active treatment of IDH may be beneficial in reducing the likelihood of experiencing composite cardiovascular events.

However, a stratified meta-analysis conducted by Jacobsen et al. [33], utilizing the 2017 ACC/AHA IDH definition, did not consistently demonstrate a connection between IDH and CVD. Moreover, any potential association observed was found to have a relatively minor impact.

Monitoring ambulatory diastolic blood pressure (DBP) and mean arterial pressure (MAP) parameters significantly improves the prediction of morbid events in individuals under 60. Conversely, for those over 60, ambulatory pulse pressure (PP) and systolic blood pressure (SBP) parameters emerge as the strongest predictors, with no additional insights from DBP or MAP values [38, 39]. Indeed, previous research has shown that as individuals’ age, an increase in arterial stiffness, coupled with a decline in DBP, is associated with the progression of atherosclerotic disease [40–42]. A 5 mm Hg rise in diastolic blood pressure correlates with a 4% higher risk of cardiovascular events, a 2% higher risk of coronary heart disease, a 3% higher risk of stroke, and a 2% higher risk of all-cause mortality [43].

Chrysant et al. [44] discussed several studies examining the relationship between isolated diastolic hypertension (IDH) and cardiovascular disease (CVD). It indicated an elevated risk of heart failure, stroke, myocardial infarction (MI), and CVD mortality with DBP in the range of 80–89 mmHg. However, concerns exist regarding the aggressive treatment of IDH, particularly in older individuals. This concern arises from the potential risks associated with excessively lowering diastolic blood pressure, attributed to a potential J-curve effect [35], which may result in adverse cardiovascular outcomes. This consideration is especially pertinent given that myocardial perfusion occurs during the diastolic phase of the cardiac cycle and relies on maintaining a minimum DBP level.

In general, although evidence indicates a link between elevated diastolic blood pressure (DBP) and negative cardiovascular outcomes, caution is warranted when considering aggressive treatment of isolated diastolic hypertension (IDH), particularly in older individuals. There is a pressing need to raise awareness about IDH. Treatment decisions should be personalized, and excessively lowering DBP, especially below 70 mmHg, may not be advisable for older subjects. This complexity emphasizes the importance of a balanced approach to managing IDH to reduce cardiovascular risks without introducing potential harm.

### Limitations

While this analysis provides valuable insights into the correlation between IDH and cardiovascular outcomes, several limitations must be acknowledged. Many outcomes exhibit high *I*^2^ values, indicating significant heterogeneity among the studies. This variability may arise from differences in study populations, methodologies, and the degree of adjustment for confounding factors. Additionally, the presence of publication bias in some analyses could influence the overall interpretation, despite efforts to address it through trim-fill analysis. Publication bias can either overestimate or underestimate the true effect size, while heterogeneity suggests that the studies may not be directly comparable due to differences in methodology or population characteristics. Although the analysis aimed to obtain the highest attainable fully adjusted risk estimate, it is important to note that the adjusted variables may not align precisely among the included studies, potentially impacting the outcomes of this investigation.

## Conclusions

This extensive analysis illuminates the varied outcomes of IDH as per different guidelines. While consistent patterns emerge across various cardiovascular and stroke-related outcomes, it is crucial to consider nuances such as study design and potential biases. In general, utilizing a higher diastolic blood pressure for IDH diagnosis revealed higher risk of CVD and outcomes. These findings underscore the significance of standardized guidelines and robust study designs to ensure accurate and meaningful insights into the effects of hypertension on health outcomes. A patient-focused approach, taking into consideration age, sex, and diagnostic criteria, has the potential to better modulate CVD outcomes in patients with IDH. Further research is warranted to delve deeper into these associations and to account for potential confounders that could influence the observed relationships. As a result, there is a clear imperative for clinical trials to evaluate the impact of antihypertensive medications and patients demographics on IDH.

## Data Availability

Yes.

## Abbreviations

IDH: Isolated diastolic hypertension
CVD: Cardiovascular disease
CHD: Coronary heart disease
AHA/ACC: American Heart Association/American College of Cardiology
JNC: Joint National Committee on Prevention, Detection, Evaluation, and Treatment of High Blood Pressure
NICE/ESC: National Institute for Health and Care Excellence/European Society of Cardiology
